# Saccadic eye movements in different dimensions of schizophrenia and in clinical high-risk state for psychosis

**DOI:** 10.1186/s12888-019-2093-8

**Published:** 2019-04-08

**Authors:** Ilya Obyedkov, Maryna Skuhareuskaya, Oleg Skugarevsky, Victor Obyedkov, Pavel Buslauski, Tatsiana Skuhareuskaya, Napoleon Waszkiewicz

**Affiliations:** 1 Republican Research and Practice Center for Mental Health, Dolginovsky Tract, 152, 220053 Minsk, Belarus; 2 Department of Psychiatry and Medical Psychology, Belarusian State Medical University, Dolginovsky Tract, 152, 220053 Minsk, Belarus; 3 Department of Psychiatry, Medical University of Bialystok, Białystok, Plac Brodowicza 1, 16-070 Choroszcz, Poland

**Keywords:** Schizophrenia, Dimensions, Psychosis high risk, Saccade, Oculography

## Abstract

**Background:**

Oculomotor dysfunction is one of the most replicated findings in schizophrenia. However the association between saccadic abnormalities and particular clinical syndromes remains unclear. The assessment of saccadic movements in schizophrenia patients as well as in clinical high-risk state for psychosis individuals (CHR) as a part of schizophrenia continuum may be useful in validation of saccadic movements as a possible biomarker.

**Methods:**

The study included 156 patients who met the ICD-10 criteria for schizophrenia: 42 individuals at clinical high-risk-state for psychosis and 61 healthy controls. The schizophrenia patients had three subgroups based on the sum of the global SAPS and SANS scores: (1) patients with predominantly negative symptoms (NS, *n* = 62); (2) patients with predominantly positive symptoms (PS, *n* = 54) (3) patients with predominantly disorganization symptoms (DS, *n* = 40). CHR subjects were characterized by the presence of one of the groups of criteria: (1) Ultra High Risk criteria, (2) Basic Symptoms criteria or (3) negative symptoms and formal thought disorders. Horizontal eye movements were recorded by using videonystagmograph. We measured peak velocity, latency and accuracy in prosaccade, antisaccade and predictive saccade tasks as well as error rates in the antisaccade task.

**Results:**

Schizophrenia patients performed worse than controls in predictive, reflexive and antisaccade tasks. Oculomotor parameters of NS were different from the other groups of patients. Latencies of predictive and reflexive saccades were significantly longer than in controls only in the NS group. The accuracy of predictive saccades was also different from controls only in the NS schizophrenia group. More prominent loss of accuracy of reflexive saccades was found in the DS group and it significantly differed from the one in other groups. Participants from DS group made more errors in antisaccade task compared to NS and PS groups. CHR subjects performed worse than controls as measured by the accuracy of reflexive saccades and antisaccades.

**Conclusions:**

The study confirms the existence of different relations between the symptom dimensions of schizophrenia and saccades tasks performances. Saccadic abnormalities were revealed in the clinical (schizophrenia) and pre-clinical (clinical high risk) populations that provide further evidence for assessing saccadic abnormalities as a possible neurobiological marker for schizophrenia.

## Background

Schizophrenia is а severe mental disorder which usually begins in adolescence and often has a chronic deteriorating course. Despite of more than a hundred years of studying, schizophrenia still is one of the most challenging problems in psychiatry. Social and economic burden related to schizophrenia is tremendous [1]. There is no full understanding of underlying processes and causal mechanisms of this disorder. Diagnosis of schizophrenia is based on the detection of clinical symptoms and depends on the clinician’s interpretation of patients’ subjective experience [2]. Researchers are trying to find more objective tests (biomarkers) to detect schizophrenia, to make a prognosis, to predict response to treatment. [3–6]. Some potential biomarkers for schizophrenia have been proposed, such as markers associated with inflammation, immune processes and with metabolic disorders or neuroendocrine/neurotrophin/ neurotransmitter alterations [5–7], as well as abnormalities in neural activity [5, 8].

Eye movement impairments have been recognized in schizophrenia patients since the early 1900s [9]. Oculomotor dysfunction is one of the most replicated findings in schizophrenia. These include impaired smooth pursuit and abnormal performance on antisaccade tasks [10]. Eye movement paradigms, especially saccadic paradigms, are useful tools in understanding neurophysiological mechanisms involved in schizophrenia [11, 12]. Saccades can be measured precisely and with a number of reliable parameters. Saccades can be categorized as visually guided saccades (reflexive saccades which are triggered exogenously and scanning saccades which are triggered endogenously), antisaccades, memory guided saccades and predictive saccades [13, 14]. Reflexive saccades or prosaccades are the most simple type of eye movements that redirect gaze, where the eye moves from a fixation point to a target. In antisaccade task upon a presentation of a peripheral target the eye must look in the equal yet opposite direction of the target [15]. The ability to look away from the target requires the inhibition of a reflexive saccade that would normally be generated in response to a visual stimulus. Generation and control of prosaccades involves subcortical (striatum, thalamus, superior colliculus, and cerebellar vermis) and cortical (primary visual, extrastriate, and parietal cortices, and frontal and supplementary eye fields) structures [16]. The same regions were activated during more complex saccades (e.g., anti-saccades, saccade sequencing). Implementation of these types of saccades requires more complex cognitive processes like inhibition and working memory, and recruits additional neural regions (such as prefrontal and anterior cingulate cortices).

Some previous schizophrenia studies have found that there are no identifiable deviations in the velocity, latency to initiation, and amplitude of reflexive saccades in schizophrenia patients regardless of medication status and chronicity as elicited by regular visually guided saccade paradigms [17–19]. However, the conclusion of normal reflexive saccade parameters has been questioned by some authors [20, 21]. Impaired performance in the predictive saccades test has previously been reported in schizophrenia patients [22, 23]. Antisaccade tasks are commonly used to explore inhibitory control, and poor inhibition has been linked with poor impulse control, agitation, excitement and hostility, as well as impairment in the dorsolateral prefrontal cortex [24, 25]. The antisaccade task generates the most frequently observed volitional-saccade abnormality in schizophrenia [26]. Increased rate of errors in saccadic task is one of the most replicable findings in schizophrenia research [15].

The study rationale has two important aspects. The first one is the dimensional approach, which proposed the independence of several psychopathological dimensions that differently relate to structural and functional systems of the brain, to the prognosis and to the treatment response [27–30]. Three-factor model of schizophrenia has been proposed by Liddle P.F. (1987) and includes factors of 1) negative symptoms (‘psychomotor poverty’), 2) positive symptoms (‘reality distortion’) and 3) thought-and-speech disorder factor (‘disorganisation’) [27–30]. To our knowledge, only one eye movement research has been conducted in relation to schizophrenic symptoms using the dimensional model [31], which revealed specific patterns of smooth pursuit abnormalities in disorganization syndrome.

The second aspect of study rationale is the assessment of oculomotor dysfunction in the broader schizophrenia continuum or in different stages of schizophrenia, which may be useful in validation of saccadic movements as a possible neurobiological marker for schizophrenia. Some saccadic research has been conducted in relation to subjects at clinical high-risk state for psychosis [32, 33], the results were heterogeneous. The definition of psychosis high risk state evolved from the application of schizophrenia prodrome concepts, and presumes that individuals so identified have high conversion rate to full-blown psychosis [34, 35].

In this study we have tested saccadic performance in different schizophrenia dimensions. We used the three-syndrome model of schizophrenia, and assessed positive, negative dimensions and disorganization. We also studied saccadic performance in the group of clinical high risk of psychosis individuals as part of schizophrenia continuum. Our hypothesis was that eye movement would be altered differently in different clinical groups. It was predicted that saccadic performance in terms of reflexive, predictive and anti-saccades would be impaired in schizophrenia patients and possibly in CHR. We also predicted that in the frame of dimensional model of schizophrenia NS and DS would be associated with saccadic abnormalities more closely than PS.

## Methods

### Participants

**Fig. 1:**
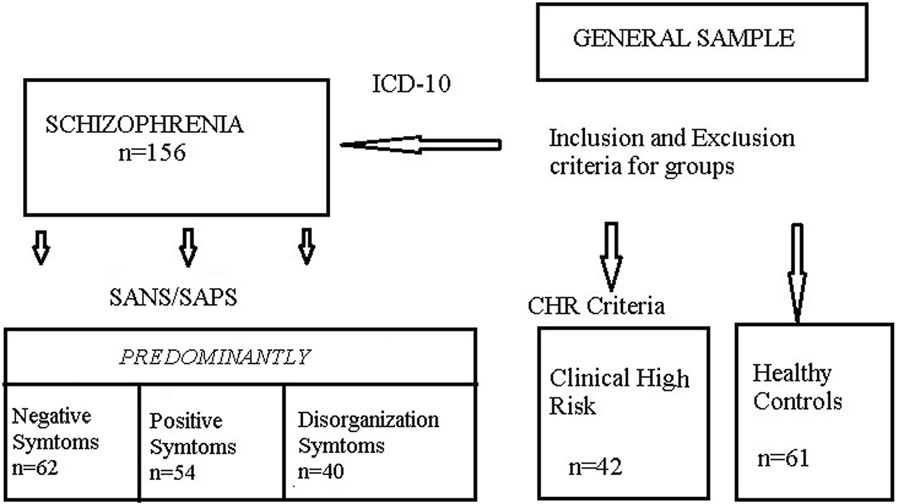
Sample selection process

The study included 156 patients who met the ICD-10 criteria for schizophrenia (SCH), 42 CHR individuals and 61 healthy controls. Sample selection process is presented in Fig. 1. Collection of data was performed consecutively during Jan 2014 – Dec 2016. All schizophrenia patients received treatment with antipsychotics at the Republican Research and Practice Center for Mental Health. The CHR subjects were recruited for this study during the medical check-up before the conscription in the same institution, they were antipsychotic free. Healthy control group was represented by sex and age matched volunteers from hospital staff. Members of the control group did not have a history of psychotic disorders or first-degree relatives with psychotic disorders. Exclusion criteria for all subjects included neurological hard signs, history of stroke and head injury, presence of drug induced extrapyramidal disorders, visual impairment other than refractive errors. All participants were examined by ophthalmologist.

Written informed consent was obtained from all participants. The study was approved by the ethic committee of the Republican Research and Practice Center for Mental Health.

The schizophrenia patients were clinically evaluated using the Scale for the Assessment of Negative Symptoms (SANS, N.Andreasen, 1983) [36] and the Scale for the Assessment of Positive Symptoms (SAPS, N.Andreasen, 1984) [37]. According to the three-syndrome model of schizophrenia [28, 31] the patients were divided into three groups based on the sum of the global SAPS and SANS scores: (1) Patients with predominantly negative symptoms (NS) (SANS global summary score ≥ 15); (2) patients with predominantly positive symptoms (PS) (SAPS score “hallucination” ≥ 6 or SAPS score “delusion” ≥ 6); (3) patients with predominantly disorganization symptoms (DS) (SAPS global score “positive formal thought disorder” ≥ 3). Patients were in a stable phase of their disease, showing no clear florid symptoms. Patients who could be attributed into more then one group were excluded from the study.

CHR subjects were characterized by the presence of one of the groups of criteria: (1) Ultra High Risk criteria [38], (2) Basic Symptoms criteria (COPER and COGDIS) [39] and (3) negative symptoms and formal thought disorders (FTD). The presence of FTD in clinical high risk individuals was assessed with methods of experimental pathopsychology. Such instruments as ‘classifications of objects and words’, ‘exclusion of objects’, ‘interpretation of metaphors’, ‘mediated memorization’ and ‘pictograms’ were used. All these instruments are widely used as a basic and well validated method of pathopsychological assessment in Russian-speaking countries [40, 41].

### Procedure

Horizontal eye movements were recorded using portable equipment - videonystagmograph ICS Chartr 200 VNG/ENG (Otometrics, Denmark). This is a complete diagnostic system for recording, analyzing, and reporting eye movements using video imaging technology, in which hi-tech video goggles with infrared cameras are used. Visual stimulus was presented on the portable light bar for oculomotor tests. Subjects were seated comfortably in a quiet, sound- and light-attenuated darkened room. A headrest was used to inhibit head movements. The stimulus was a red light that was presented at the light emitting diode bar 80 cm away from the head. Before each trial, the system was calibrated by asking the subject to look at the center and ± 10°. Every single recording was visually inspected on the computer monitor and blinks and artifacts were cut out.

We used three saccadic tasks:

1. Predictive saccades. Two targets + 15° and - 15° of the midline appeared alternatively at a fixed frequency. In this task we assessed mean latency, peak velocity and accuracy, and did not select anticipatory saccades.
2. Reflexive saccades (prosaccades). After central fixation a peripheral target appeared at the light bar pseudo-randomly. The range of target amplitude is 5 degrees to 30 degrees.
3. Antisaccades. A visual stimulus is presented and participants are required to make an eye movement away from the target to its mirror position.

For the first two of these tasks subjects were instructed to fixate the target as quickly and accurately as possible whenever it changed its position. At least 60 saccades for every paradigm were analyzed per each participant. Saccadic eye movements were analyzed in terms of velocity (maximum velocity reached in a saccadic movement as measured over an 18.75 ms period), accuracy (amplitude of the patient’s saccade divided by the amplitude of target movement expressed in percent), and latency (time between stimulus movement and the first eye movement of more than 108 degrees/second). In antisaccades task we assessed the errors (the percentage of the wrong antisaccades) as the most reliable measure of antisaccade performance [42]. Applied algorithm rejects artifacts in eye movements that occur too early (250 ms before and 75 ms after target movement), too late (the default is more than 600 ms after target has moved), or in the wrong direction.

### Statistical analysis

All analysis was performed using the Statistical Package for the Social Sciences (IBM SPSS Statistcs 20). The Kolmogorov-Smirnov test was performed to assess the normality of distribution. Demographic data was compared using independent samples t-test and chi-square tests for categorical data. We used the three-step approach to compare saccadic performance: (1) comparison between subjects with schizophrenia and healthy controls; (2) comparison between schizophrenia and CHR subgroups; (3) post-hoc pairwise comparisons if *p*-value from ANOVA was significant (*p* < 0.05). Fisher’s least significant difference (LSD) tests have been performed in ANOVA post-hoc analysis. Correlations between different saccadic tasks have been examined with Pearson correlation coefficients.

## Results

Demographic and clinical characteristics of the groups are shown in Table 1.

**Table 1:** Demographic and clinical characteristics of the groups; CHR -high risk of psychosis individuals, NS -patients with predominantly negative symptoms, PS -patients with predominantly positive symptoms, DS -patients with predominantly disorganization symptoms

Groups	n	Male (%)	Age (years)	Duration of illness (years)
SCH	156	71 (45.5)	34.4 ± 11.8	9.88 ± 8.74
NS	62	30 (48.3)	33.3 ± 11.2	8.7 ± 3.4
PS	54	23 (42,6)	36.89 ± 11.6	11.3 ± 6.9
DS	40	21 (52.5)	32.3 ± 12.2	10.53 ± 4.8
CHR	42	42 (100)	21.8 ± 6.3	N/A
Control	61	28 (45.9)	36.4 ± 11.4	N/A

CHR group members were younger than the other four groups, with no difference between the other four groups. All CHR subjects were males.

Comparison of schizophrenia patients and controls revealed no difference in velocity of reflexive (451.85, SD = 39.35 in control, 454.84, SD = 40.28 in SCH; *p* > 0.05) and predictive saccades (446.44, SD = 40.01 in control, 453.26, SD = 64.6 in SCH; *p* > 0.05). Latencies of predictive saccades were longer in schizophrenia patients compared to controls (250.83, SD = 32.32 in control, 273.24, SD = 64.93 in SCH; *p* < 0.05) as well as latencies of reflexive saccades (242.53, SD = 23.99 in control, 265.56, SD = 65.68 in SCH; *p* < 0.01). Accuracies were significantly higher in the control group in reflexive (95.11, SD = 3.17 in control, 76.46, SD = 15.03 in SCH; *p* < 0.0001) and predictive saccades (93.98, SD = 3.23 in control, 86.73, SD = 16.10 in SCH; *p* < 0.001). In the antisaccade task participants with schizophrenia performed worse than controls and made more errors (28.85, SD = 7.46 in schizophrenia group; 10.3, SD = 6.14 in control group, t = 17.420, *p* < 0.001).

In the general group of participants we found correlations between the error rates in antisaccade task and: latencies of predictive saccades (*r* = 0.223, *p* < 0.001), accuracy of predictive saccades (*r* = − 0.298, *p* < 0.001) and accuracy of reflexive saccades (*r* = − 0.311, *p* < 0.001). In the general group of participants latencies of predictive saccades highly correlated with their accuracies (*r* = − 0.704, *p* < 0.001). The correlation of reflexive saccade latency with their accuracy was weak (*r* = − 0.152, *p* < 0.05). When correlation analysis was applied in different groups, such a tendency was present only in NS (*r* = − 0.754, *p* < 0.001 for predictive task and *r* = − 0.302, *p* < 0.01 for reflexive task).

Means, standard deviations (SDs), ANOVA results and significance values of differences between controls and all other groups for predictive and reflexive saccade tasks are shown in Table 2.

**Table 2:** ANOVA results for predictive and reflexive saccades tasks; CHR -high risk of psychosis individuals, NS -patients with predominantly negative symptoms, PS -patients with predominantly positive symptoms, DS -patients with predominantly disorganization symptoms

	Predictive saccades			Reflexive saccades		
	Latency (ms)	p	Velocity (degees/sec)	Latency (ms)		Velocity (degees/sec)
PS	250.7 ± 34.3	0.274	459.9 ± 34.5	233.0 ± 29.0	0.353	462.4 ± 35.9
NS	285.5 ± 73.1	0.001	451.6 ± 52.8	283.2 ± 78,9	0.000	447.3 ± 39.7
DS	251.5 ± 31.6	0.937	447.9 ± 33.4	243.3 ± 35.7	0.885	457.4 ± 46.0
CHR	247.7 ± 35.5	0.811	461.3 ± 46.2	231.8 ± 58.3	0.223	462.1 ± 40.8
Control	251.6 ± 32.7		455.9 ± 36.3	242.3 ± 23.8		452.3 ± 34.0
ANOVA	*F* = 11.31, *p* = 0.000		*F* = 0.61, *р* = 0.65	*F* = 6.68, *p* = 0.000		*F* = 0,11; *p* = 0.977

No difference was found in velocity of predictive and reflexive saccades between clinical groups and controls. Mean peak velocities in all groups were close to physiological normal values. The post hoc LSD test showed that latencies of predictive and reflexive saccades were longer in the NS than in controls. PS, DS and CHR did not differ in terms of latencies from controls.

**Fig. 2:**
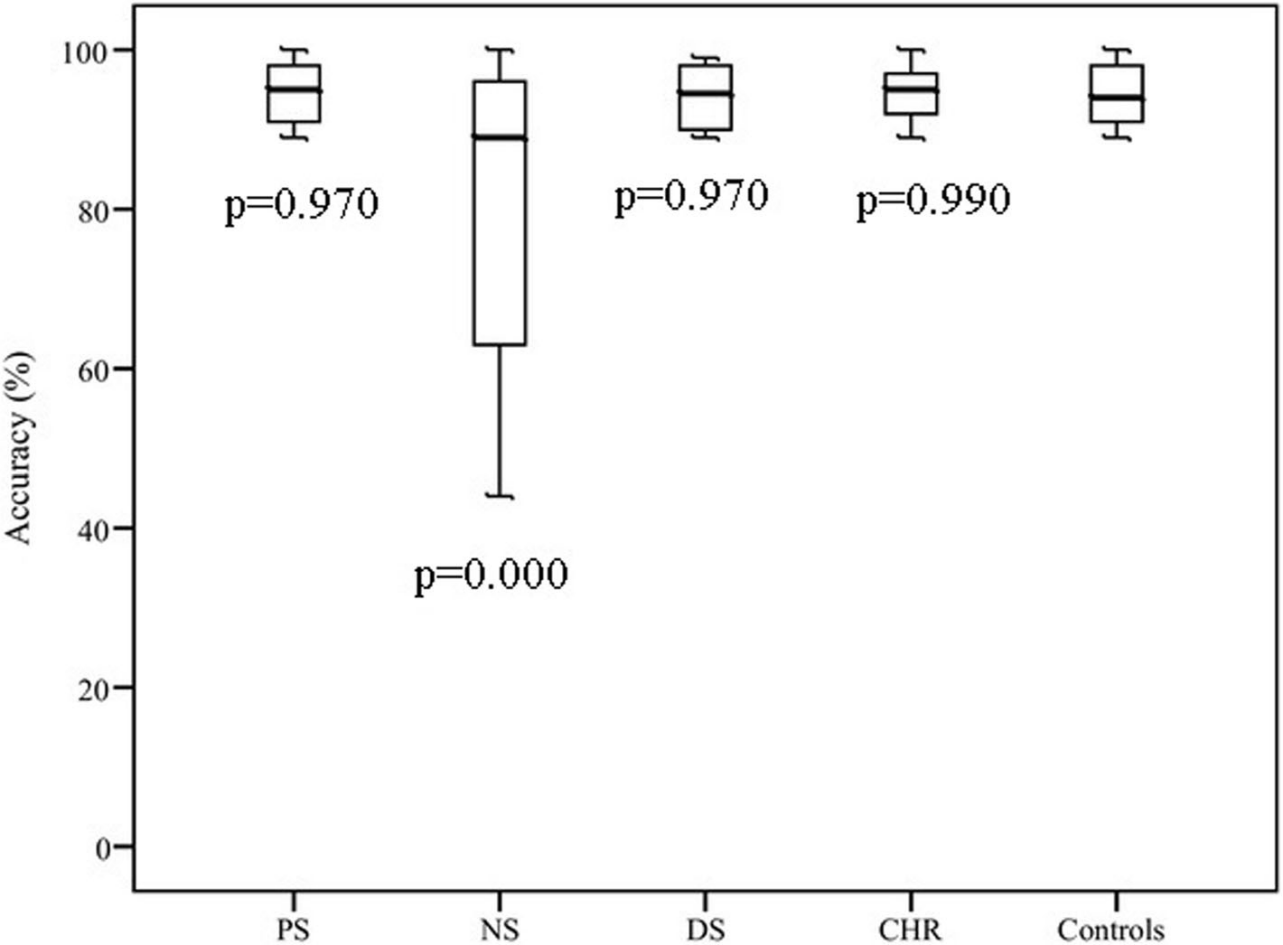
Accuracy of predictive saccades with *p*-values for post-hoc LSD test for each subgroup compared to healthy controls; CHR -high risk of psychosis individuals, NS –patients with predominantly negative symptoms, PS -patients with predominantly positive symptoms, DS-patients with predominantly disorganization symptoms

**Fig. 3:**
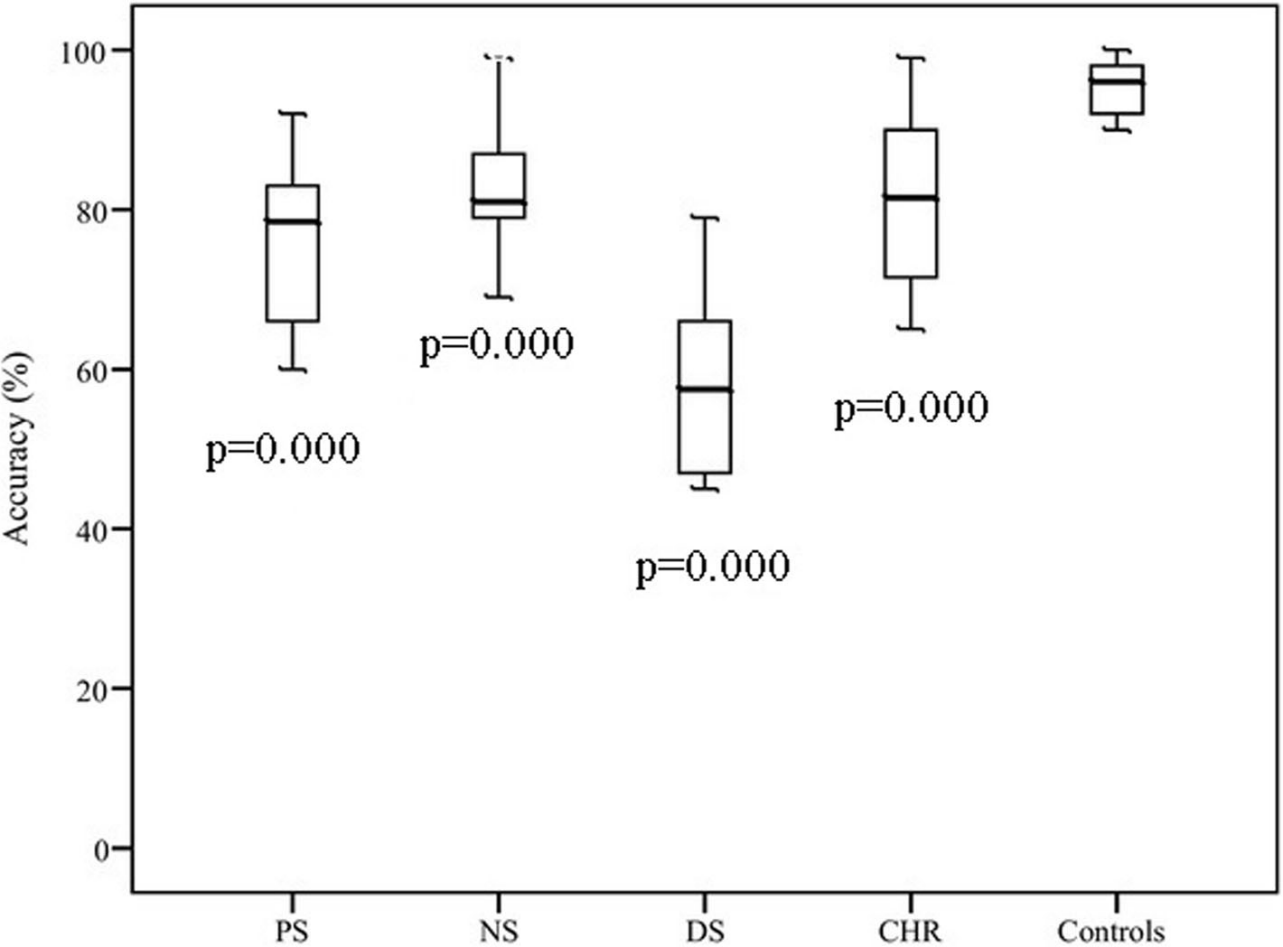
Accuracy of reflexive saccades with *p*-values for post-hoc LSD test for each subgroup compared to healthy controls; CHR -high risk of psychosis individuals, NS –patients with predominantly negative symptoms, PS -patients with predominantly positive symptoms, DS-patients with predominantly disorganization symptoms

ANOVA showed a significant group effect of accuracy of predictive (*F* = 14.54, *p* = 0.000) and reflexive (*F* = 48.53, *p* = 0.000) saccades. Results are shown in Figs. 2 and 3.

Post-hoc LSD test reveales that in predictive saccade task NS (*p* = 0.000) performed worse in terms of accuracy than controls. We did not find any difference in accuracy of predictive saccades between PS, DS, CHR and controls.

In reflexive saccades task accuracies in all schizophrenia groups and CHR group were worse than in controls. More prominent loss of accuracy of reflexive saccades was found in the DS group (60%) and it significantly differed from the one in other groups (post-hoc LSD test, *p* < 0.001). The results of CHR was close to the results of NS group (80%).

**Fig. 4:**
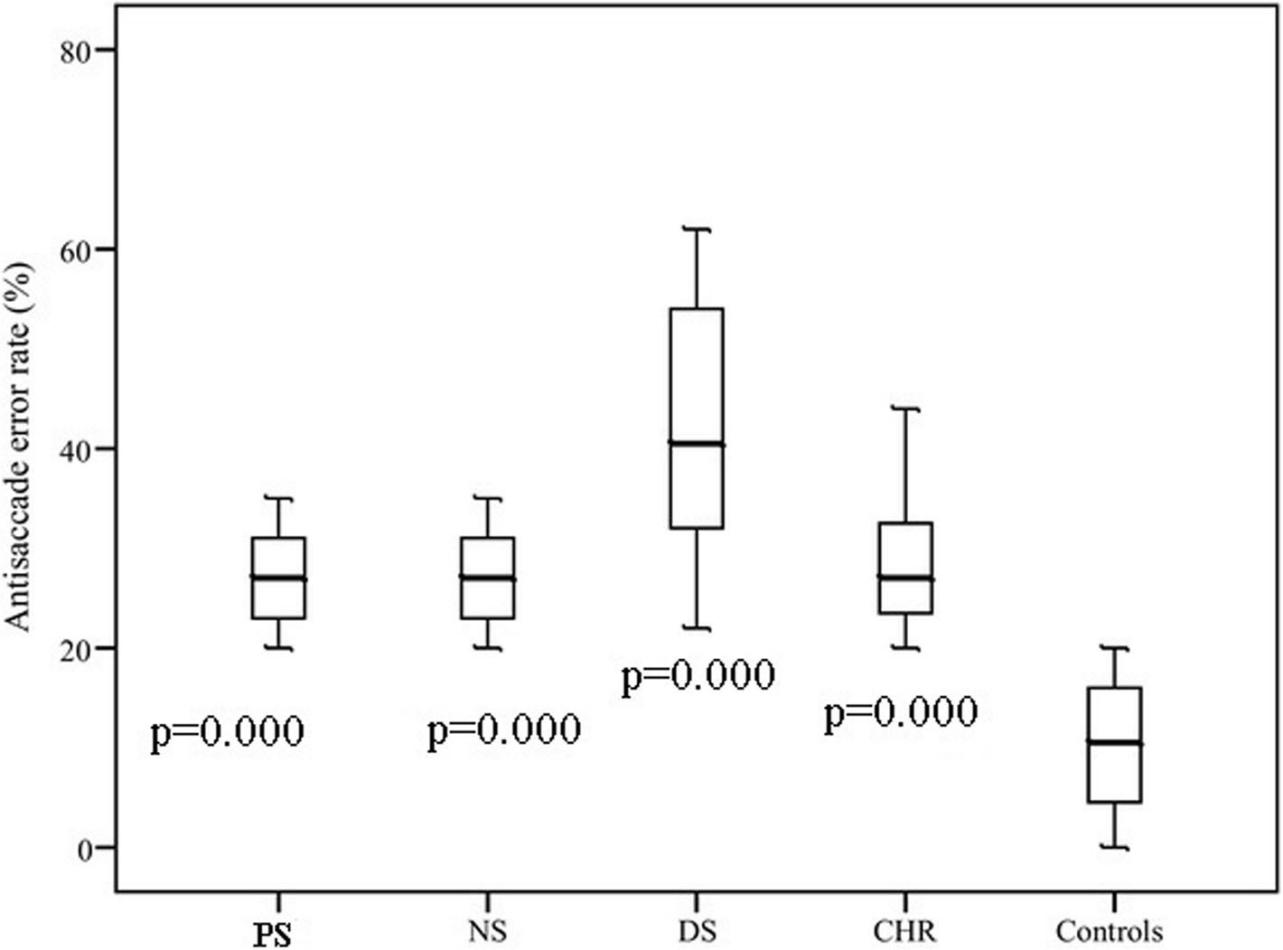
Antisaccade errors with p-values for post-hoc LSD test for each subgroup compared to healthy controls; CHR -high risk of psychosis individuals, NS -patients with predominantly negative symptoms, PS -patients with predominantly positive symptoms, DS -patients with predominantly disorganization symptoms

ANOVA revealed a significant group effect of antisaccade errors (*F* = 96.79, *p* = 0.000). The results of antisaccade task (antisaccade errors) in different schizophrenia groups, CHR and control groups are presented in Fig. 4.

The post hoc LSD test showed that all schizophrenia groups and CHR group made more errors in antisaccade task than controls. DS group performed worse than all other groups. The performance of CHR was close to PS and NS.

## Discussion

The results of this study support our initial hypothesis that the main syndromes of schizophrenia are accompanied by specific patterns of saccadic abnormalities. Oculomotor parameters of NS were different from the other groups of patients. Latencies of predictive and reflexive saccades were significantly longer than in controls only in the NS group. The accuracy of predictive saccades was also different from controls only in the NS schizophrenia group. Our results are consistent with those of other researchers who found motor, cognitive and neuropathological differences in patients with prominent negative symptoms when compared to patients who do not have prominent negative symptoms [43–45]. The neural basis for negative symptoms (apathy, poverty of speech, emotional blunting, avolition etc.) is the cortico-striato-thalamo-cortical circuit dysfunction including predominantly prefrontal cortex dysfunction [46–48]. The dorsolateral prefrontal cortex (DLPFC) is known for its involvement in executive functions (such as working memory, cognitive flexibility, planning, inhibition and etc.) [49–51] deterioration of which is the primary feature of schizophrenia. The DLPFC is involved in ocular motor behavior, i.e. saccadic eye movements, especially intentional saccades [52–55]. As it has been mentioned by Pierrot-Deseilligny et al. [52], DLPFC is located at a strategic place between the attentional (posterior cingulate cortex) and motivational (anterior cingulate cortex) areas, the executive cortical (frontal eye field and supplementary eye field) and brainstem (superior colliculus) ocular motor areas. Wide involvement of the prefrontal cortex in pathological processes in NS patients may result in saccadic movement abnormalities even in a simple task.

In reflexive saccades with a random visual stimulus, test accuracies of all schizophrenia groups were worse than in the control group. Minimal accuracy was found in the DS group. Neurophysioligical specificity of the DS group (prolonged latency of P300 wave of the evoked cognitive auditory potentials, impaired executive neuropsychological functioning in the executive sphere) was previously reported by Nestsiarovich et al. [56]. The loss of accuracy in reflexive saccade test instead of predictive saccade test may be explained by a more difficult task of targeting a random signal rather than targeting a predictively appearing signal. In our study we used sequences of tasks – firstly with the predictable appearance of the stimulus than with the random appearance of the stimulus. The change to the task with unpredictible stimuli might be challenging for patients. It was previousely reported by Nieuwenhuis et al. [57] that schizophrenia patients displayed normal performance on the simple version of the prosaccade task. However, when administered a more difficult version of a prosaccade task, schizophrenia patients performed significantly worse than controls [57]. Latencies compared to accuracies were more closely associated with negative symptoms of schizophrenia. Prolonged latencies in NS appeared in different types of tasks (predictive or reflexive) and so, latencies may be considered sensitive markers of negative sumptoms of schizophrenia.

It appeared that latencies and accuracies of reflexive saccades did not highly correlate. The significant correlations for predictive and reflexive saccades were found only in NS. It may be explained by complexity of eye movement regulation and by non-linear relationship between different types of saccadic parameters.

In agreement with previous reports [17, 58], the results revealed more errors in antisaccade task in all schizophrenia groups than in controls. Antisaccade errors reflect failures of response suppression [15]. Our results support the idea of inadequate response inhibition as a core executive function deficit in schizophrenia. Participants from DS group made more errors in antisaccade task compared to NS and PS groups. Different relations between the symptom dimensions of schizophrenia and deficit in executive functioning were previously reported by several studies [59, 60]. Disorganization symptoms were associated with attentional dysfunction and with failure to suppress inappropriate responses [59] and our results confirm it.

CHR group differs from controls in accuracy of reflexive saccades and antisaccades. Only several studies have investigated performance on saccadic tasks in clinical high risk populations. By contrast to Caldini study [32], we found that the error rate in antisaccade task was significantly higher in CHR compared to controls. The performance on antisaccade task in CHR was close to the one of schizophrenia patients. So, our results support that eye movement alterations are possible markers of clinical high risk of psychosis. It may be the result of dorsolateral prefrontal cortex dysfunction. Several studies have reported cognitive impairment in CHR subjects [61–64], which is predominantly focused on executive functions and working memory. These cognitive functions and voluntary saccades performance are associated with DLPFC. Abnormal antisaccade task performance is present at the early stages of schizophrenia and reflects working memory and inhibitory control disturbances. Taking into account the fact that CHR group participants did not receive antipsychotic treatment, we can propose that the eye movement alterations are present at the early stages of schizophrenia and do not depend on treatment. Although in order to prove that the assessment of antipsychotic-naïve patients with schizophrenia is needed.

Accuracy of eye movements is controlled by a ‘brain error detector’, which is localized predominantly in anterior cingulate cortex, eye fields of the frontal and supplementary motor cortex areas [65]. The brain error detector can detect and correct signals from proprioreceptors of external eye muscles, from photoreceptors of the retina and efferent copy signals. The worse performance on the saccade task in schizophrenia patients compared to controls may be explained by the brain error detector failure or by the corollary discharge (CD) dysfunction, a mechanism for distinguishing self-generated percepts from externally generated percepts [66–68]. The main idea of CD in eye movements is that the brain informs and modulates the activity of sensory processing systems of eye position via an efference copy of motor command, CD signal allows frontal eye field to update receptive field to the new target location before the execution of a saccade [69]. CD failure results in deficits of self-monitoring and inability of schizophrenia patients to accurately attribute sensory stimulus as internally- or externally-generated. Hallucinations and delusions are closely associated with CD dysfunction.

### Limitations

The limitations of this study include the absence of sample size calculation for selected groups, which makes the evaluation of the statistical power adequacy difficult. Differences in terms of eye movement recording techniques (videonistagmography or electonistagmography), as well as differences in oculomotor tests used have been posited to account for the possible difficulties in comparing our results with the results of other studies in the field. Potential confounding effect of antipsychotic treatment may be important. There is a possibility of oculomotor changes in the course of treatment.

## Conclusions

The study confirms the existence of different relations between the symptom dimensions of schizophrenia and saccades tasks performances. According to the three- dimensional model of schizophrenia negative syndrome but not positive or disorganized syndromes was associated with latencies of predictive and reflexive saccades. Patients with predominantly disorganization syndrome demonstrate more prominent loss of accuracy of the reflexive saccades as compared to others groups. Saccadic abnormalities were revealed in clinical (schizophrenia) and pre-clinical (clinical high risk) populations that provide further evidence for assessing saccadic abnormalities as a possible neurobiological marker for schizophrenia.

Further research should examine the replicability of our findings and consider the effect of additional potential confounders such as antipsychotic treatment, age, gender on the relationship between eye movements and clinical representations. Application of different eye movements tests such as smooth pursuit eye movement or optokinetic test may support our results. Further research requires detailed assessment of schizophrenia endophenotypes with inclusion of first-degree relatives of schizophrenia patients.

## Abbreviations

CHR: Clinical high risk of psychosis
DS: Disorganization symptoms
FTD: Formal thought disorders
ICD-10: 10th revision of the International Statistical Classification of Diseases and Related Health Problems
NS: predominantly negative symptoms
PS: Predominantly positive symptoms
SANS: The Scale for the Assessment of Negative Symptoms
SAPS: The Scale for the Assessment of Positive Symptoms
